# Perceived Stress and Daily Well-Being During the COVID-19 Outbreak: The Moderating Role of Age

**DOI:** 10.3389/fpsyg.2020.571873

**Published:** 2020-11-12

**Authors:** Da Jiang

**Affiliations:** ^1^Department of Special Education and Counselling, Education University of Hong Kong, Tai Po, Hong Kong; ^2^Centre for Psychosocial Health, Education University of Hong Kong, Tai Po, Hong Kong; ^3^Integrated Centre for Wellbeing (I-WELL), Education University of Hong Kong, Tai Po, Hong Kong

**Keywords:** affective experiences, perceived stress, age, daily diary, COVID-19

## Abstract

**Objectives:**

Older adults are considered one of the most vulnerable groups to COVID-19. However, previous studies on emotion and aging have found that older adults report better wellbeing than younger adults in global surveys and daily reports. To better understand older adults’ wellbeing during the COVID-19 outbreak, we examined age differences in daily affective experiences in this study.

**Methods:**

Two hundred and thirty-one participants from mainland China aged 18 to 85 were recruited to participate in the 14-day daily diary study, after a pretest. Their trait affect and demographic information were measured in the pretest. Their daily affect and stress levels were measured in the daily assessments.

**Results:**

I found that older adults reported lower perceived stress related to COVID-19 in daily life, compared to younger adults. The negative relationship between daily perceived stress and high arousal positive affect and the positive relationship between daily perceived stress and high arousal negative affect was weaker in older than younger adults.

**Discussion:**

These results provide initial evidence of daily affective wellbeing across different age groups in adulthood during the COVID-19 outbreak. Such information is important for developing interventions to promote better wellbeing during the COVID-19 outbreak.

## Introduction

The Coronavirus Disease 2019 (COVID-19) epidemic started in December 2019 and quickly spread to more than 215 countries by mid-April 2020. Due to its serious effects on human health, it can also damage mental health by increasing levels of anxiety, stress, and worry among health professionals, and the general public (Qiu et al., 2020). Although older adults are considered one of the most vulnerable groups to COVID-19 (Remuzzi and Remuzzi, 2020), some global surveys found that older adults reported less depression and anxiety (Bruine de Bruin, 2020; Losada-Baltar et al., 2020). However, few studies so far have examined whether the daily wellbeing of older adults was indeed better than their younger counterparts. To this end, I examined the daily perceived stress of Chinese people across different age groups in adulthood during the COVID-19 outbreak. In addition, I examined age differences in the relationship between daily perceived stress and daily wellbeing.

In the literature on aging and emotion, older adults generally report a higher level of positive affect (e.g., happiness, enthusiastic, and calm) and a lower level of negative affect (e.g., anxiety, sadness, and stress) than younger adults in global surveys and daily diary studies (Gross et al., 1997; Carstensen et al., 2000). These results have been explained by socioemotional selectivity theory (SST) (Carstensen et al., 2003). SST postulates that older adults prioritize emotionally meaningful goals (e.g., experience positive emotions) over knowledge goals (e.g., learn new knowledge) more than younger adults, because they view future time as more limited. Prioritizing emotionally meaningful goals, older adults are more motivated to regulate their emotions toward a positive end (Fung and Carstensen, 2006). Therefore, they report better wellbeing in general even after experiencing negative affect. In a laboratory experiment, Scott et al. (2017) found no age difference in regulating momentary negative stressors (0–10 min), but older adults better regulated stressors after being exposed to stressors for 10 min to 2.5 h. The COVID-19 outbreak is a situation that induces tremendous psychological distress (Qiu et al., 2020). Although older adults reported better mental health in one-off surveys (Bruine de Bruin, 2020; Yang et al., 2020), it is unknown whether older adults can still regulate their emotions better than younger adults in real-time daily life. Therefore, I examined age differences in daily perceived stress and its relationship with daily wellbeing using a 14-day daily diary study in a lifespan sample.

Based on the two-dimensional valence-arousal model (Russell, 1980), I examined people’s daily affective experiences during the COVID-19 outbreak using a daily diary method by which participants’ affective experiences were measured on a daily basis. In particular, I focused on daily stress, and daily experiences of the positive affect (POS), high arousal positive affect (HAP), low arousal positive affect (LAP), negative affect (NEG), high arousal negative affect (HAN), and low arousal negative affect (LAN). Based on SST (Carstensen et al., 2003), I predicted that older adults would experience a lower level of perceived stress related to COVID-19 (H1). In addition, I predicted that the relationship between daily perceived stress and daily wellbeing (indexed by higher levels of POS, HAP, and LAP, and lower levels of NEG, HAN, and LAN) would be weaker in older than younger adults.

## Materials and Methods

### Participants

Two hundred and thirty-one Chinese participants aged 18 to 85 (*M*_*age*_ = 44.74 years, *SD*_*age*_ = 17.54 years; 69% women; 70% had a college degree; 41% had a job; 17% had a religion) participated in the study in February 2020. Thirty-three percent of the sample were aged between 18 to 35 years, 51.5% were aged between 36–60 years, and 15% were aged between 61 years and above. They were recruited through mass mailing through a university email system. All participants were born and raised in China, and lived in mainland China during the 14-day daily diary period. They came from 23 of the 32 provinces, cities, and autonomous regions in mainland China. Two participants came from Hubei province. We included their data in the analysis because the results did not change when deleting their data. However, four participants were excluded from the data analysis due to missing data in their daily diaries. Among the remaining 227 participants, 192 completed 14 daily questionnaires (*Range* = 1–14 times; *M*_*assessment*_ = 13.26, *SD* = 2.66). One of them was identified as a confirmed or suspected COVID-19 case during the questionnaire period, but the deletion of the data did not change the pattern of the results. Descriptive information about the sample is presented in Table 1.

**TABLE 1:** Descriptive statistics of all study variables.

	Minimum	Maximum	Mean	SD
Age	18.00	85.00	44.74	17.54
Overall health	1.00	6.00	4.19	0.93
Subjective socioeconomic status	2.00	10.00	6.20	1.61
Daily HAP*	1.00	5.00	3.00	0.98
Daily LAP*	1.00	5.00	2.94	0.84
Daily HAN*	1.00	5.00	1.90	0.90
Daily LAN*	1.00	5.00	1.92	0.99
Daily stress*	1	5.00	2.32	0.75
Gender (female%)			69%	
Marital%			13%	
Education (% college)			71%	
Religion (% have a religion)			17%	

*N* = 231. HAP = high-arousal positive affect; LAP = low-arousal positive affect; HAN = high-arousal negative affect; LAN = low-arousal negative affect. * indicates weighted means by the number of assessments.

### Procedure

The study was conducted during the peak period of COVID-19 spread in China. All questionnaires were completed online using the Wenjuan.com online survey system. After an introductory e-mail and a briefing session via WeChat, the participants were asked to complete an online survey on their demographic information. The daily diary period started from the second day after the first online survey and lasted 14 consecutive days. A WeChat message containing the URL link to the online questionnaire was sent to the participants around 8 pm each day to remind them to complete the daily questionnaire. They received another reminder via WeChat if they had not completed the questionnaire by 9 am the next morning. All participants received HK$200 (approximately US$25) after completing the study. This study was approved by the Human Research Ethics Committee of the Education University of Hong Kong.

### Measures

#### Trait Questionnaire

##### Actual trait affect

We used the Affect Valuation Index (AVI; Tsai et al., 2006) to measure their actual trait affect. The participants were asked to indicate how often they actually experienced each affective state in a typical week on a 5-point scale, ranging from 1 “*never*” to 5 “*always.*” Based on the two-dimensional valence-arousal model, HAP was measured by the aggregate score of “enthusiastic,” “excited,” and “elated” (Cronbach’s α = 0.68); LAP was measured by the aggregate score of “calm,” “relaxed,” “peaceful,” and “serene” (Cronbach’s α = 0.76); HAN was measured by the aggregate score of “fear,” “hostile,” and “nervous” (Cronbach’s α = 0.71); and LAN was measured by the aggregate score of “dull,” “sleepy,” and “sluggish” (Cronbach’s α = 0.71).

##### Demographic information

The participants were asked to indicate their gender (0 = male, 1 = female), partner status (0 = without partner, 1 = with partner), education (0 = did not finish college, 1 = finished college), religion (0 = no religion, 1 = has a religion), and overall subjective health (1 = very poor to 6 = perfect).

#### Daily Questionnaire

##### Daily actual affect

The daily version of the AVI (Tsai et al., 2006) was used to assess daily actual affect. The participants were asked to indicate the intensity with which they actually experienced each affective state on that day on a 5-point scale, ranging from 1 “*not at all*” to 5 “*extremely*.” In particular, HAP was measured by “enthusiastic” (intraclass correlation coefficients (ICC) = 53%); LAP was measured by “calm” (ICC = 44%); HAN was measured by “anxious” (ICC = 52%); LAN was measured by “dull” (ICC = 55%); positive affect was measured by “happy” (ICC = 51%); and negative affect was measured by “sad” (ICC = 49%).

##### Perceived stress related to COVID-19

The Perceived Stress Scale (Cohen et al., 1983) was adapted to measure the participants’ daily perceived stress related to COVID-19. “In the last month” in the original version was changed to “today” to measure daily stress. Three items for unexpected life changes were used in the short daily questionnaire, including “I was upset because of COVID-19 today,” “I felt that I was unable to control the important things in my life because of COVID-19 today,” and “Despite COVID-19, I felt confident about my ability to handle my personal problems.” The participants were asked to indicate how often they agreed with the statement, from 1 “*never*” to 5 “*always*” (ICC = 67%, between-person reliability estimate = 0.71; within-person reliability estimate = 0.68; Cranford et al., 2006).

##### Subjective health

The participants were asked to rate their daily subjective health on a scale from 1 “*very poor*” to 6 “*perfect.*”

## Data Analysis and Results

Age differences in perceived daily stress during the COVID-19 outbreak were first examined. Then whether there were age differences in the relationship between daily stress and daily affective experiences were determined. These questions were addressed using Hierarchical Linear Modeling (HLM; Raudenbush, 2004). The results were controlled for daily subjective health, the number of days of assessment, marital status, socioeconomic status, education, and religion because these variables were found to be associated with daily affect and stress. Marital status, religion, and education were included in the model as bivariate variables, while age, daily subjective health, and socioeconomic status were centered on the grand mean. HLM models of the two research questions are reported below.

### HLM Model Equations

#### Age Differences in Daily Perceived Stress

Below are the model equations addressing this question.

Level-1 model

**Perceived stress** = B0 + B1^∗^(Day) + B2^∗^(daily health) + r

Level-2 model

B0 = G00 + G01^∗^(age) + G02^∗^(marital status) + G03^∗^(socioeconomic status) + G04^∗^ (education) + G05^∗^(religion) + u0
B1 = G10 + u1
B2 = G20 + u2

#### The Relationship Between Daily Affective Experiences and Perceived Stress

Level-1 model

**Affective experiences** = B0 + B1^∗^(day) + B2^∗^(daily health) + B3^∗^(daily perceived stress) + r

Level-2 model

B0 = G00 + G01^∗^(gender) + G02^∗^(age) + G03^∗^(marital status) + G04^∗^(socioeconomic status) + G05^∗^(education) + G06^∗^(religion) + u0
B1 = G10 + u1
B2 = G20 + u2
B3 = G30 + u3

#### Age Differences in Daily Perceived Stress

With perceived stress as the dependent variable, the variance components (VC) suggested that there was significant variance in the intercept to be explained across individuals (χ*^2^* = 1020.62, *p* < 0.001). Age was associated with daily lesser perceived stress (*estimate* = −0.006, *SE* = 0.003, *p* < 0.05). Ordinal day was associated with less positive affect, (*estimate* = −0.010, *SE* = 0.003, *p* < 0.01), suggesting that participants reported less stress in the later days of the daily diary period. Detailed results are reported in Table 2.

**TABLE 2:** Multilevel hierarchical linear analysis testing the age differences in daily perceived stress.

	Estimate	SE
**Level 1**		
Day	0.006	0.003
Daily health	−0.082**	**0.023**
Level 2		
Intercept	1.801**	**0.173**
Age	−0.006*	**0.003**
Marital	−0.151	0.124
SES	−0.059*	**0.025**
Education	−0.106	0.106
Religion	−0.226*	**0.111**

**Random effects**	**Variance component**	**χ*^2^***

Intercept	**0.604**	1020.62**
Day	**0.001**	450.81**
Health	**0.046**	374.82**

*N* for level 1 variables is 3030, and *N* for level 2 variables is 222. * *p* < 0.05, ** *p* < 0.001.

#### The Relationship Between Daily Affective Experiences and Perceived Stress

Age differences in the relationship between perceived stress and daily affective experiences were then examined. Perceived stress was negatively associated with daily positive affect (*estimate* = −0.282, *SE* = 0.033 *p* < 0.001), HAP (*estimate* = −0.153, *SE* = 0.035, *p* < 0.001), and LAP (*estimate* = −0.078, *SE* = 0.034, *p* < 0.05); and was positively associated with daily negative affect (*estimate* = 0.247, *SE* = 0.029, *p* < 0.001), HAN (*estimate* = 0.431, *SE* = 0.032, *p* < 0.001), and LAN (*estimate* = 0.302, *SE* = 0.033, *p* < 0.001). The relationship between stress and HAP was moderated by age so that the negative association was weaker in older than younger adults (*estimate* = 0.005, *SE* = 0.002, *p* < 0.05). The relationship between stress and HAN was also weaker in older than younger adults (*estimate* = −0.005, *SE* = 0.002, *p* < 0.05). Age did not moderate the relationships between stress and other daily affective states. Detailed results are reported in Table 3.

**TABLE 3:** Multilevel hierarchical linear analysis testing the age differences in the relationship between perceived stress and daily affective experiences.

	Positive		Negative		HAP		LAP		HAN		LAN	
	Estimated	SE	Estimated	SE	Estimated	SE	Estimated	SE	Estimated	SE	Estimated	SE
**Level 1**												
Day	−0.013*	**0.004**	−0.013*	**0.004**	0.003	0.005	−0.026***	**0.004**	−0.006	0.004	**−0.018**	**0.004**
Daily health	0.132***	**0.026**	−0.064*	**0.024**	0.140***	**0.029**	0.020	0.024	−0.108***	**0.027**	−0.121***	**0.030**
Stress	−0.282***	**0.033**	0.247***	**0.029**	−0.153***	**0.035**	−0.078*	**0.034**	0.431***	**0.032**	0.302***	**0.033**
**Level 2**												
Intercept	3.388***	**0.089**	1.582***	**0.068**	2.955***	**0.109**	3.117***	**0.087**	2.061***	**0.077**	2.194***	**0.092**
Age	−0.001	0.003	−0.000	0.002	0.001	0.003	0.003	0.003	−0.009***	**0.002**	−0.005	0.003
Marital	0.014	0.116	−0.018	0.082	−0.255	0.142	−0.200	0.113	0.070	0.099	−0.012	0.120
SES	0.074*	**0.025**	−0.044*	**0.017**	0.040	0.030	−0.009	0.024	−0.008	0.021	−0.071*	**0.025**
Education	−0.064	0.101	0.023	0.071	0.005	0.123	0.063	0.098	−0.142	0.086	−0.151	0.104
Religion	0.122	0.106	0.009	0.069	0.142	0.129	−0.127	0.104	0.040	0.088	−0.132	0.106
**L1 X L2 Interaction**												
Stress X Age	0.003	0.002	0.002	0.002	0.005*	**0.002**	0.003	0.002	−0.005*	**0.002**	0.001	0.002
Random effects	VC	χ*^2^*	VC	χ*^2^*	VC	χ*^2^*	VC	χ*^2^*	VC	χ*^2^*	VC	χ*^2^*
Intercept	**0.270**	362.433***	**0.216**	360.014***	**0.684**	469.96***	**0.238**	324.001***	**0.207**	358.153***	**0.272**	384.803***
Day slope	**0.002**	256.632***	**0.002**	267.800***	**0.002**	352.46***	0.001	179.337	**0.002**	309.785***	**0.002**	284.361***
Daily health Slope	**0.037**	218.230**	**0.0352**	287.927***	**0.043**	0.043*	0.019	179.065	**0.051**	272.732***	**0.059**	264.771***
Stress slope	**0.058**	**175.876**	**0.051**	205.276*	0.050	183.36	**0.067**	210.44*	**0.056**	251.472***	**0.046**	198.94*

*N* for level 1 variables is 3030, and *N* for level 2 variables is 222. HAP = high-arousal positive affect; LAP = low-arousal positive affect; HAN = high-arousal negative affect; LAN = low-arousal negative affect. *p < 0.05, **p < 0.01, ***p < 0.001.

## Discussion and Conclusion

Using a 14-day daily diary study, we examined people’s daily affective experiences across different age groups in adulthood during home quarantine when COVID-19 broke out in mainland China. We found that older adults reported a lower level of perceived stress related to COVID-19 than younger adults. In addition, we found that the negative association between daily perceived stress and daily high arousal positive affect was weaker in older than younger adults; and that the positive relationship between daily perceived health and daily high arousal negative affect was also weaker in older than younger adults.

Consistent with the previous findings on age differences in perceived stress after stress induction (Scott et al., 2017), we found that older adults reported a lower level of daily perceived stress than younger adults during the COVID-19 outbreak. These findings could be explained by SST that found that older adults are more motivated to regulate emotion than younger adults (Carstensen et al., 2003). In addition, these results are consistent with a recent one-off surveys on age differences in loneliness during home quarantine in Spain (Losada-Baltar et al., 2020) and China (Qiu et al., 2020). These findings, taken together, may suggest that older adults indeed perceived less stress during the outbreak of COVID-19.

We only found significant age differences in the relationship between daily stress and high arousal affect, regardless of valence. Such findings may be attributable to the fact that stress is a high arousal affective state. Therefore, the feeling of stress might be more relevant to high arousal positive and negative affect than the low arousal ones. It may also be possible that individuals regardless of age prefer low arousal affect states over high arousal affect states, when they see future time as increasingly limited (Jiang et al., 2016). The situation of COVID-19 induces a more limited future time perspective, because it damages both mental and physical health (Yang et al., 2020). Low arousal affective states are more relevant to the situation of COVID-19 for both younger and older adults. Therefore, the age differences were only observed in high arousal affective states. Future studies should clarify the mechanism of the age differences in the relationship between daily stress and different types of daily affective states. Although older adults are considered as one of the most vulnerable groups during COVID-19, the findings of this study, together with the previous findings, may suggest that older adults indeed have a better ability to cope with the psychological distress caused by COVID-19. However, more support and effort should be given to protect their physical health (Nikolich-Zugich et al., 2020).

Despite its interesting results, the study also has limitations. First, although we included multiple assessment points, this study was based on a correlational design. Thus, we were not able to examine the mechanism underlying the relationship. Second, the sample used may not be representative of the population of mainland China. In particular, the education level in this sample was higher than that of the general population. Although demographic information, such as education level, marital status, religion, and socioeconomic status, was controlled for in data analysis, a larger sample size is needed to validate findings of this initial study on daily affective experiences during the COVID-19 outbreak in future studies. Third and as aforementioned, we could not explain the non-significant interactions between age and fate control on daily affective experiences. Future studies should clarify this issue.

In this 14-day daily diary study, we found that older adults reported a lower level of perceived stress related to COVID-19 than younger adults. The negative relationship between perceived stress and a high arousal positive affective state, and the positive relationship between perceived stress and a high arousal negative affective state were weaker in older than younger adults. These results provide initial evidence of the daily affective wellbeing of adults during the COVID-19 epidemic in China. These findings provide initial evidence on daily affect during home quarantine of individuals across different age groups in adulthood. Such information may be important for preparing different mental health services for people in different age groups.

## Data Availability Statement

The raw data supporting the conclusions of this article will be available upon sending request to the corresponding author, without undue reservation.

## Ethics Statement

The studies involving human participants were reviewed and approved by the Human Research Ethics Committee of the Education University of Hong Kong. The patients/participants provided their written informed consent to participate in this study.

## Author Contributions

The author contributes to theoretical framework, data analysis, and manuscript writing of the manuscript.

## Conflict of Interest

The author declares that the research was conducted in the absence of any commercial or financial relationships that could be construed as a potential conflict of interest.
